# Unbalanced Hybrid AOA/RSSI Localization for Simplified Wireless Sensor Networks

**DOI:** 10.3390/s20143838

**Published:** 2020-07-09

**Authors:** Anh Tuyen Le, Le Chung Tran, Xiaojing Huang, Christian Ritz, Eryk Dutkiewicz, Son Lam Phung, Abdesselam Bouzerdoum, Daniel Franklin

**Affiliations:** 1 School of Electrical, Computer and Telecommunications Engineering, University of Wollongong, Wollongong 2522, Australia; atl102@uowmail.edu.au (A.T.L.); critz@uow.edu.au (C.R.); phung@uow.edu.au (S.L.P.); a.bouzerdoum@uow.edu.au (A.B.); 2 School of Electrical and Data Engineering, University of Technology Sydney, Ultimo 2007, Australia; xiaojing.huang@uts.edu.au (X.H.); Eryk.Dutkiewicz@uts.edu.au (E.D.); Daniel.Franklin@uts.edu.au (D.F.); 3 Division of Information and Computing Technology, College of Science and Engineering, Hamad Bin Khalifa University, Doha 34110, Qatar

**Keywords:** angle of arrival (AOA), received signal strength indicator (RSSI), target localization, wireless sensor network (WSN), positioning

## Abstract

Source positioning using hybrid angle-of-arrival (AOA) estimation and received signal strength indicator (RSSI) is attractive because no synchronization is required among unknown nodes and anchors. Conventionally, hybrid AOA/RSSI localization combines the same number of these measurements to estimate the agents’ locations. However, since AOA estimation requires anchors to be equipped with large antenna arrays and complicated signal processing, this conventional combination makes the wireless sensor network (WSN) complicated. This paper proposes an unbalanced integration of the two measurements, called 1AOA/nRSSI, to simplify the WSN. Instead of using many anchors with large antenna arrays, the proposed method only requires one master anchor to provide one AOA estimation, while other anchors are simple single-antenna transceivers. By simply transforming the 1AOA/1RSSI information into two corresponding virtual anchors, the problem of integrating one AOA and *N* RSSI measurements is solved using the least square and subspace methods. The solutions are then evaluated to characterize the impact of angular and distance measurement errors. Simulation results show that the proposed network achieves the same level of precision as in a fully hybrid nAOA/nRSSI network with a slightly higher number of simple anchors.

## 1. Introduction

Global positioning system (GPS) has made our daily lives much more convenient due to the tracking function available in mobile phones, cars, watches, etc. With GPS receivers equipped in such devices, their movements in outdoor areas can be accurately located and tracked, providing vast benefits in navigation and management. However, since at least four visible GPS satellites are required to locate a receiver’s position, the performance of GPS-based systems becomes inaccurate in indoor environments, dense urban areas, or deep forests [1]. To improve accuracy, the combination between GPS and opportunistic terrestrial signals, such as Wi-Fi, has been explored in the literature, e.g., [2]. In emergency scenarios, such as fire, post-tsunami, post-earthquake, and military missions however, these signals may be unavailable, sabotaged, or even denied. Hence, a wireless sensor network (WSN) must be deployed to provide the localization services for first responders.

Typically, a WSN includes a number of anchors, whose locations are known, and other unknown nodes, called agents. In a range-based WSN, anchors receive signals from unknown nodes, perform ranging measurements, and send information to a center node which estimates agents’ locations [3]. There are many different techniques to obtain ranging information based on time-of-arrival (TOA), time-different-of-arrival (TDOA), time-of-flight (TOF), angle-of-arrival (AOA), and received signal strength indicator (RSSI). The more accurate the ranging information is, the more precise the locations of agents can be estimated. However, the accuracy of ranging measurements depends on the complexity of the anchors. For example, distance estimation based on TOA, TDOA, and TOF can be very accurate, but it requires strict synchronization among anchors and agents [4]. Therefore, a fundamental problem of localization in WSNs is to precisely locate the agents, while the complexity and power consumption of the anchors are minimized.

Among various approaches in the literature, hybrid AOA/RSSI methods have been proven to be very effective [5]. Since RSSI is always available in receivers, using RSSI for localization is one of the most popular methods [6]. Using path loss models, if we know the transmitted power of an agent, its distances to anchors can be determined from RSSI. These distances can be used in a lateration method to draw multiple circles, then their intersections are used to localize the agent. However, as RSSI is affected by multipath, noise, and shadowing, precision of standalone RSSI positioning is low. Meanwhile, if there exist line-of-sight paths from agents to anchors, AOA estimation can provide the directions toward the unknown nodes. Combination of RSSI and AOA, therefore, can significantly reduce the estimation error [7].

An example of combining RSSI and AOA localization can be found in Guideloc systems proposed in [8] for disaster rescue applications. In this system, unmanned aerial vehicles (UAVs) are used to search the positions of the targets. The RSSI and AOA received by the UAVs are combined to control the trajectories of the UAVs to fly over the targets. Thereby, the targets’ locations can be derived from the GPS coordinates of the UAVs. Since both RSSI and AOA measurements are utilized, the search time can be reduced significantly.

In other applications such as localization and tracking of first responders, RSSI and AOA information is collected by the anchors and processed at a data fusion center. In this case, target positioning using hybrid AOA/RSSI data can be cast as a nonconvex optimization problem [9]. Solutions of this problem can be categorized into optimization-based and least square (LS) estimators [10]. Examples of the former approaches can be found in [10,11] using a maximum likelihood (ML) estimator. While the ML algorithm achieves great accuracy, it suffers from severe computational complexity and the global solution is not guaranteed [6]. To reduce complexity while obtaining a comparable accuracy to ML, semidefinite programming and second-order cone programming relaxation techniques have been proposed in [12,13,14,15]. However, the computational complexity of these algorithms grows significantly with the network size [5]. In comparison, the LS estimators are less accurate, but they are more computationally efficient. Therefore, great efforts have been devoted to improve their accuracy by applying a weighting matrix to the LS solution, so called weighted LS (WLS). In [9,16,17,18], a weighting covariance matrix is derived based on noise statistics. However, since noise statistics are not available, they need to be estimated first. As a result, they are not suitable for real-time applications due to the delay occurring in the noise estimation process. Another way to construct the weighting matrix is to utilize the distances among the anchors and the agents as proposed in [19]. It was proved that both RSSI and AOA short-range measurements are more reliable than the long ones. Hence, emphasis should be given to the shorter links. As shown in [5], this WLS estimator achieves remarkable precision, while its complexity is comparable to LS method.

The above-mentioned hybrid AOA/RSSI localization methods assume that AOA and RSSI measurements are available at all anchors. However, AOA estimation is a challenging task which normally requires an antenna array and complicated signal processing. For example, in [20], two perpendicularly orientated directional antennas are employed and the RSSI from these antennas are fitted into parabolic functions to estimate AOA. Otherwise, a large antenna array with multiple signal classification algorithms must be equipped to obtain AOA information [6]. Obviously, such a WSN requiring AOA estimation at every anchor leads to significant hardware complexity and power consumption. Especially in applications such as emergency responses and disaster rescues, which require flexible and fast deployment of WSNs, anchors can be carried by UAVs [8,21]. Therefore, requiring every anchor to be equipped with an antenna array is not practical.

In this paper, an unbalanced hybrid AOA/RSSI localization method, called 1AOA/nRSSI, is proposed to simplify the complexity of the WSN. In particular, LS algorithms are employed to estimate the unknown nodes based on 1 AOA and *n* RSSI measurements (n≥3). As a result, the WSN in this case only needs a master anchor which is equipped with an antenna array to perform AOA estimation, while other anchors are simple single-antenna transceivers to acquire RSSIs. Simulations are then conducted to show that, with a slightly higher number of simple anchors, the root mean square error of the proposed method is equivalent or superior to that obtained by a fully equipped WSN performing the 3AOA/3RSSI WLS algorithm. Meanwhile, under a significant shadowing environment, the proposed 1AOA/nRSSI method approaches the precision of a 3AOA/3RSSI network if more simple anchors are deployed. The contributions of this paper are summarized as follows. Firstly, unbalanced hybrid AOA/RSSI localization is proposed to reduce the burden of AOA estimation in all anchors of the WSN. Secondly, by simply transforming one AOA information into two virtual anchors with corresponding ranging measurements, we present two algorithms, namely, LS and subspace estimators. The impact of measurement errors on the performance of these two algorithms is evaluated. Finally, simulation results show that the proposed 1AOA/nRSSI approach is promising in low shadowing environments, for example, from a UAV to the ground agents.

The remainder of this paper is organized as follows. Section 2 introduces system models and some related works. Section 3 presents the proposed 1AOA/nRSSI estimator with two different algorithms. Section 4 provides the evaluation results of the two algorithms under different simulation scenarios. Finally, Section 5 concludes the paper. Note that a list of abbreviations used in this paper is given at the end of the paper.

## 2. Related Works

Consider a WSN including *N* anchors whose locations are known at $a_{i}=\left[a_{ix},a_{iy}\right],i=1,\ldots ,N$, and *M* agents whose actual locations are $x_{m}=\left[x_{mx},x_{my}\right],m=1,\ldots ,M$. In this paper, we only present the results for a two-dimensional map for the ease of illustration. At the anchor $a_{i}$, the RSSI $P_{i}$ and AOA $\alpha_{i}$ measured from the *m*-th agent are expressed as

(1) $$\begin{array}{cc} P_{i} & =P_{0}-10\gamma \log_{10}\left(\frac{∥x_{m}-a_{i}∥}{r_{0}}\right)+n_{i}\\ \alpha_{i} & =\tan^{-1}\left(\frac{x_{my}-a_{iy}}{x_{mx}-a_{ix}}\right)+m_{i}\;\mathrm{for}\;i=1,\ldots ,N, \end{array}$$

where $P_{0}$ is the received power at the reference distance $r_{0}$, γ is the path-loss exponent (PLE), ∥.∥ denotes the Euclidean norm, $n_{i}$ is the log-normal shadowing, and $m_{i}$ is the angle estimation noise. Both $n_{i}$ and $m_{i}$ are modeled as zero-mean Gaussian random variables with standard deviations $\sigma_{n_{i}}$ (dB) and $\sigma_{m_{i}}$ (degree), respectively—i.e., $n_{i}∼N\left(0,\sigma_{n_{i}}^{2}\right),m_{i}∼N\left(0,\sigma_{m_{i}}^{2}\right)$. The problem here is to estimate the location of the agent $x_{m}$ based on $P_{i}$ and $\alpha_{i}$. Some typical localization methods are presented as follows.

### 2.1. RSSI Localization

RSSI is used to determine the distance $r_{i}$ from the agent to the *i*-th anchor. Assuming PLE is known at the anchor, the distance $r_{i}$ can be determined by

(2) $$r_{i}=r_{0}\;10^{\frac{P_{0}-P_{i}+n_{i}}{10\gamma }}.$$

The localization problem becomes solving a set of nonlinear equations of two variables $x_{mx},x_{my}$ expressed as

(3) $${\left(x_{mx}-a_{ix}\right)}^{2}+{\left(x_{my}-a_{iy}\right)}^{2}=r_{i}^{2},\;\mathrm{for}\;i=1,\ldots ,N.$$

The agent’s location can be found with as few as two anchors using the bilateration method [6], which plots two circles centered at the two anchors and uses one of their intersections as the agent’s position. However, due to the noise effect, the two circles may not intersect and the location cannot be found. Therefore, normally, at least three anchors are required, so Equation (3) becomes an overdetermined set of nonlinear equations. To solve this problem, it can be firstly transformed into linear equations and then solved by a linear LS solution. As presented in [22], choosing the *r*-th anchor as a reference anchor, and then subtracting the remaining equations in Equation (3) to the *r*-th one, we have a set of (N−1) linear equations as

(4) $$\left(a_{ix}-a_{rx}\right)x_{mx}+\left(a_{iy}-a_{ry}\right)x_{my}=\frac{1}{2}\left[a_{ix}^{2}+a_{iy}^{2}-a_{rx}^{2}-a_{ry}^{2}-r_{i}^{2}+r_{r}^{2}\right],\;\mathrm{for}\;i=1,\ldots ,N,i\neq r.$$

The agent’s location, denoted for this RSSI localization method as $x_{m}^{R}$, can be found by solving Equation (4) based on the LS solution as

(5) $$\begin{array}{c} {\hat{x}}_{m}^{R}={\left(H^{T}H\right)}^{-1}H^{T}b, \end{array}$$

where

$$H=\left[\begin{array}{cc} a_{1x}-a_{rx} & a_{1y}-a_{ry}\\ \vdots & \vdots \\ a_{Nx}-a_{rx} & a_{Ny}-a_{ry} \end{array}\right],b=\frac{1}{2}\left[\begin{array}{c} K_{1}^{2}-K_{r}^{2}-r_{1}^{2}+r_{r}^{2}\\ \vdots \\ K_{N}^{2}-K_{r}^{2}-r_{N}^{2}+r_{r}^{2} \end{array}\right],$$

and $K_{i}^{2}=a_{ix}^{2}+a_{iy}^{2},i=1,\ldots ,N$. The linear LS solution is simple, but its accuracy is relatively low compared to nonlinear LS method [6]. However, since a nonlinear LS is an optimization problem, its global solution may not be guaranteed while it requires a very high level of complexity.

To achieve a better accuracy at a reasonable cost of complexity, subspace localization using TOA [23] and RSSI measurements [24] is presented as follows. Assume that the WSN is fully cooperative, i.e., all the anchors and agents are in the communication ranges, or the anchors know the positions of each other. Define an N×2 matrix X as

(6) $$X=\left[\begin{array}{cc} a_{1x}-x_{mx} & a_{1y}-x_{my}\\ \vdots & \vdots \\ a_{Nx}-x_{mx} & a_{Ny}-x_{my} \end{array}\right].$$

From X, construct a multidimensional similarity matrix, denoted by D, as

(7) $$D=XX^{T}.$$

Here, $D_{i,k}$ element is

(8) $$\begin{array}{cc} D_{i,k} & =\left(a_{ix}-x_{mx}\right)\left(a_{kx}-x_{mx}\right)+\left(a_{iy}-x_{my}\right)\left(a_{ky}-x_{my}\right)\\ \; & =\frac{1}{2}\left(d_{i}^{2}+d_{k}^{2}-d_{ik}^{2}\right), \end{array}$$

where $d_{i}$ and $d_{k}$ are the real distances between the *i*-th and *k*-th anchors to the *m*-th agent, respectively, while $d_{ik}^{2}={∥a_{i}-a_{k}∥}^{2},i,k=1,\ldots ,N$. Applying the eigenvalue decomposition of D, we have

(9) $$D=U\Lambda U^{T},$$

where $\Lambda =\mathrm{diag}\{\lambda_{1},\lambda_{2},\ldots ,\lambda_{N}\}$ is the diagonal matrix of eigenvalues of D with $\lambda_{1}\geq \lambda_{2}\geq \ldots \geq \lambda_{N}$, and U is an orthonomal matrix whose columns are the corresponding eigenvectors $u_{n},n=1,\ldots ,N$. Since the rank of D is 2, $\lambda_{3}=\lambda_{4}=\ldots =\lambda_{N}=0$, therefore, Equation (9) can be written as

(10) $$\begin{array}{cc} D & =U_{s}\Lambda_{s}^{1/2}{\left(U_{s}\Lambda_{s}^{1/2}\right)}^{T}\\ \; & =U_{s}\Lambda_{s}^{1/2}\Omega {\left(U_{s}\Lambda_{s}^{1/2}\Omega \right)}^{T}, \end{array}$$

where $U_{s}=\left[u_{1}\;u_{2}\right]$, $\Lambda_{s}=\mathrm{diag}\left\{\lambda_{1},\lambda_{2}\right\}$, and Ω is an unknown rotation matrix satisfying $\Omega \Omega^{T}=I_{N}$, where $I_{N}$ denotes the identity matrix of order N. From Equations (7) and (10), we have

(11) $$X=U_{s}\Lambda_{s}^{1/2}\Omega .$$

The unknown rotation matrix Ω can be determined based on the LS rule as

(12) $$\Omega ={\left[{\left(U_{s}\Lambda_{s}^{1/2}\right)}^{T}\left(U_{s}\Lambda_{s}^{1/2}\right)\right]}^{-1}{\left(U_{s}\Lambda_{s}^{1/2}\right)}^{T}X.$$

Substituting Equation (12) into Equation (11), we obtain

(13) $$X=U_{s}U_{s}^{T}X.$$

This means that the agents’ position $x_{m}$ can be extracted from the signal subspace eigenvectors. Since D is not available, its approximation version, denoted by $\hat{D}$, can be constructed by

(14) $${\hat{D}}_{i,k}=\frac{1}{2}\left(r_{i}^{2}+r_{k}^{2}-d_{ik}^{2}\right),$$

where $r_{i}$ and $r_{k}$ are calculated based on Equation (2). Hence, Equations (12) and (13) become approximations. As a result, we have

(15) $$X\approx {\hat{U}}_{s}{\hat{U}}_{s}^{T}X,$$

where ${\hat{U}}_{s}$ is the signal subspace of $\hat{D}$. Denoting $A=\left[\begin{array}{cc} a_{1x} & a_{1y}\\ \vdots & \vdots \\ a_{Nx} & a_{Ny} \end{array}\right]$, X can be written as $X=A-1_{N}x_{m}$, where $1_{N}$ is the N×1 vector of all ones. Substituting this expression to Equation (15) and rearranging it, we have

(16) $$\left(I_{N}-{\hat{U}}_{s}{\hat{U}}_{s}^{T}\right)1_{N}x_{m}\approx \left(I_{N}-{\hat{U}}_{s}{\hat{U}}_{s}^{T}\right)A.$$

Note that $I_{N}-{\hat{U}}_{s}{\hat{U}}_{s}^{T}={\hat{U}}_{n}{\hat{U}}_{n}^{T}$, where ${\hat{U}}_{n}$ is the noise subspace of $\hat{D}$. The LS solution of Equation (16) gives the agent’s estimated location, denoted by $x_{m}^{S}$, as

(17) $$x_{m}^{S}=\frac{1_{N}^{T}{\hat{U}}_{n}{\hat{U}}_{n}^{T}A}{1_{N}^{T}{\hat{U}}_{n}{\hat{U}}_{n}^{T}1_{N}}.$$

**Figure 1:**
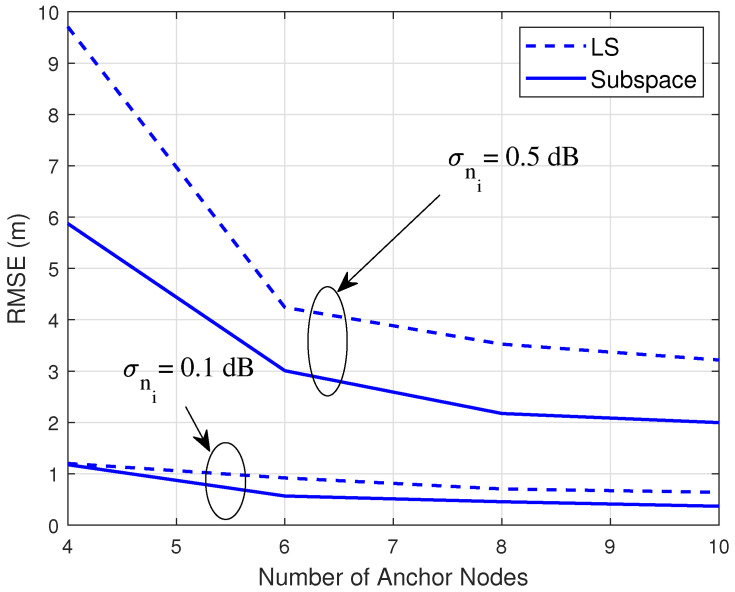
Comparison of the subspace and least square (LS) solutions.

Figure 1 compares the root mean square errors (RMSEs) of the subspace method and the LS counterpart under different levels of shadowing and number of anchors. We can see that under the same levels of RSSI errors, the subspace method outperforms the LS method, especially when a larger number of anchors is deployed.

### 2.2. Hybrid AOA/RSSI Localization

**Figure 2:**
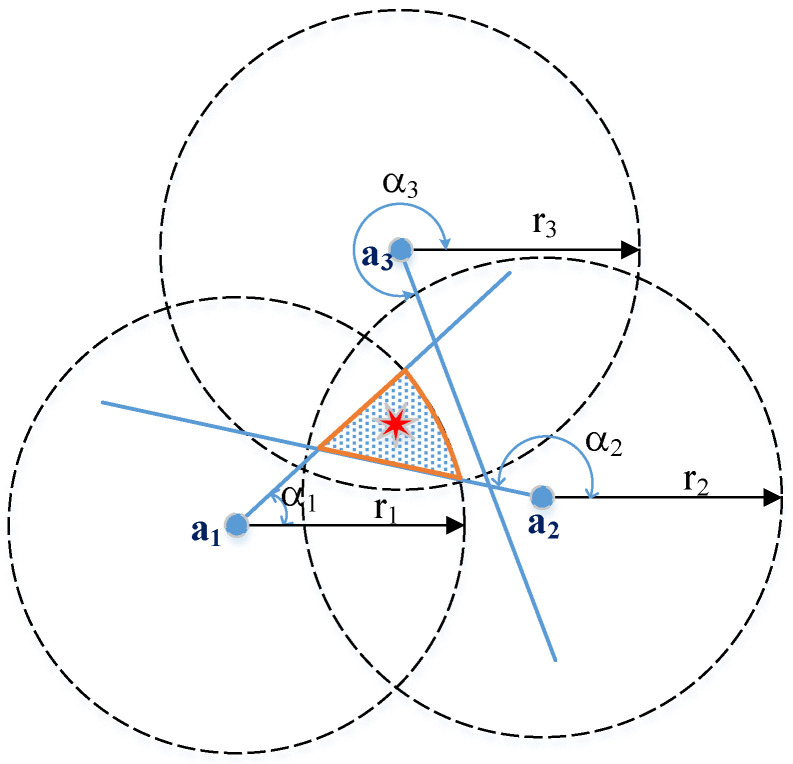
Hybrid nAOA/nRSSI localization. AOA—angle-of-arrival; RSSI—received signal strength indicator.

Due to measurement errors, standalone RSSI localization cannot precisely locate agents. Therefore, combining the range measurement with an AOA estimation can significantly improve the accuracy. As shown in Figure 2, using both RSSI and AOA information, possible location of the agent (red star) is within the shaded area, which is smaller than that defined by standalone RSSI (3 circles) and AOA (3 lines). With both AOA and RSSI available at each anchor, we have a new set of linear equations as

(18) $$\begin{array}{cc} {\hat{x}}_{mx} & =a_{ix}+r_{i}\cos\left(\alpha_{i}\right)\\ {\hat{x}}_{my} & =a_{iy}+r_{i}\sin\left(\alpha_{i}\right),i=1,\ldots ,N. \end{array}$$

Using the LS method, the solution for this problem is found [25]:

(19) $$\begin{array}{c} {\hat{x}}_{m}^{nAnR}={\left(S^{T}S\right)}^{-1}S^{T}u, \end{array}$$

where $S=\left[\begin{array}{cc} 1_{N} & 0_{N}\\ 0_{N} & 1_{N} \end{array}\right]$; $1_{N}$ and $0_{N}$ are column vectors of *N* ones and *N* zeros, respectively; and $u={\left[a_{1x}+r_{1}\cos\left(\alpha_{1}\right),\cdots ,a_{Nx}+r_{N}\cos\left(\alpha_{N}\right),a_{1y}+r_{1}\sin\left(\alpha_{1}\right),\cdots ,a_{Ny}+r_{N}\sin\left(\alpha_{N}\right)\right]}^{T}$. The accuracy of this method can be improved by applying a weight $w_{i}=1-\frac{r_{i}}{\sum_{i=1}^{N}r_{i}}$ on different links [19]. This is because a closer link normally has a smaller error than the longer one. The weighted least square (WLS) solution is expressed as

(20) $$\begin{array}{c} {\hat{x}}_{m}^{nAnR}={\left(S^{T}W^{T}S\right)}^{-1}S^{T}W^{T}u, \end{array}$$

where $W=I_{2}⊗\mathrm{diag}\left\{w\right\}$, $I_{2}$ denotes an identity matrix of order 2, ⊗ is Kronecker product, and $w=\left[\sqrt{w_{1}},\cdots ,\sqrt{w_{N}}\right]$. Due to its simplicity and high accuracy, this WLS solution, referred to as “*nAnR-WLS*”, will be used as a benchmark in this paper.

## 3. The Proposed Method

Geometrically, conventional hybrid AOA/RSSI localization aims to reduce the area where the agent possibly resides, as shown in Figure 2. However, not all of the AOA measurements contribute to the improvement of the accuracy, especially, when AOA estimation suffers from high measurement errors. Since a high-resolution AOA estimation requires complicated signal processing and a large antenna array, conventional hybrid AOA/RSSI leads to complex anchors and so does the WSN.

**Figure 3:**
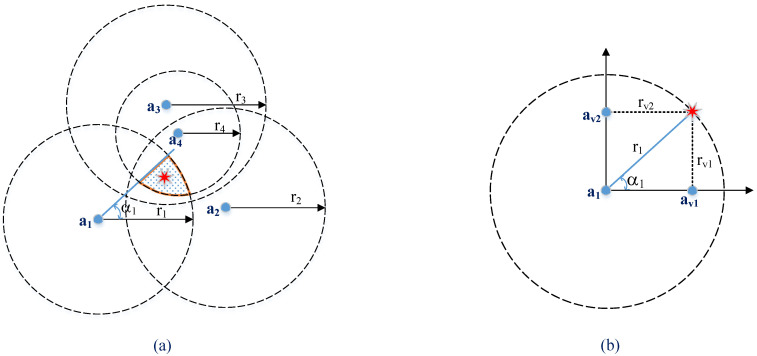
Unbalanced hybrid AOA/RSSI localization: (**a**) 1AOA/4RSSI and (**b**) Angular-to-ranging transformation.

To overcome this problem, we propose an unbalanced hybrid method, called 1AOA/nRSSI, as follows. Instead of estimating AOA from all anchors, we only use one AOA estimation to be combined with *N* RSSI measurements. As a result, in the WSN with the 1AOA/nRSSI method, only one master anchor, which is equipped with a large antenna array and powerful signal processing, is employed to obtain the AOA information. Other anchors are simple single-antenna transceivers to measure RSSI. In case a significant error is encountered in RSSI measurements, more simple anchors can be deployed to obtain the same reduction of the potential area as in the case of a fully hybrid AOA/RSSI method. This reduction is shown in Figure 3a.

There are many different ways of integrating one AOA and *N* RSSI measurements. Unlike conventional hybrid nAOA/nRSSI that enjoys *N* linear equations as in Equation (18), in the case of 1AOA/nRSSI, we only have one linear equation which is 1AOA/1RSSI, while *N* RSSI measurements are nonlinear ones. Therefore, *N* ranging measurements can be transformed into angular information, as proposed in [26]. In this transformation, given two anchors $a_{1},a_{2}$ with the ranges to the agent of $r_{1},r_{2}$, draw a line that passes the intersections of the two circles centered at $a_{1}$ and $a_{2}$ with the radii $r_{1}$ and $r_{2}$, respectively. This line is thus perpendicular to the line connecting these two anchors. Thereby, the vertices of the polygonal area in which the agent resides can be found from the ranging measurements $r_{1}$, $r_{2}$, and $d_{12}$. Then, the LS solution is applied to (N+1) linear equations. However, when the shadowing effect is significant, ranging-to-angular transformation leads to cumulative errors, yielding a lower precision.

We propose a simple method to integrate 1AOA with *N* RSSI measurements as follows. Without loss of generality, we assume that the master anchor is at the origin. From the AOA $\alpha_{1}$ and the range $r_{1}$ estimated at the master anchor, we can determine two new virtual anchors, denoted as $a_{v1},a_{v2}$, corresponding to the ranging information $r_{v1},r_{v2}$, respectively. As illustrated in Figure 3b, the virtual anchors’ locations and $r_{v1},r_{v2}$ can be calculated as

(21) $$\begin{array}{cc} a_{v1} & =[r_{1}\cos\left(\alpha_{1}\right)\;\;0]\\ a_{v2} & =[0\;\;r_{1}\sin\left(\alpha_{1}\right)]\\ r_{v1} & =r_{1}\sin\left(\alpha_{1}\right)\\ r_{v2} & =r_{1}\cos\left(\alpha_{1}\right). \end{array}$$

We now have a set of (N+2) anchors and the corresponding ranging information. From this set, LS and subspace solutions presented in Section 2.1 can be used to locate the agents. We define these methods as “*1AnR-LS*” and “*1AnR-Subspace*” in the rest of the paper.

One problem of the LS method is to select the reference anchor (cf. Equation (4)). As presented in [27], the anchor that is closest to the agent should be chosen. However, in this set of (N+2) anchors, the master anchor at the origin is used three times, i.e., once as the real anchor $a_{1}$ with the range $r_{1}$, and twice as virtual anchors $a_{vi}$ with the ranges $r_{vi},i=1,2$. This means that the performance of the 1AOA/nRSSI localization relies significantly on the master anchor, especially when the number of anchors is small. As a result, choosing the master anchor as a reference node is a good decision in case of a small number of available anchors. This interpretation will be confirmed later in our simulations. The solution that employs the master anchor as the reference is denoted as *1AnR-LS*. Its performance will be compared to that of *1AnR-LS-minRef* [27]—which is the LS solution using the closest anchor as the reference—in the next section.

## 4. Performance Evaluation and Discussions

### 4.1. Simulation Setup

Simulations are conducted using MATLAB to evaluate and compare the precision of the proposed approaches and that of the conventional nAOA/nRSSI. A WSN with *N* anchors is randomly deployed in an area of 100 m × 100 m to localize 2000 agents whose locations are also random in the area. Among *N* anchors, the first one is fixed at the origin ($a_{1}=\left[0\;0\right]$) which provides AOA estimation for the 1AOA/nRSSI method, while other anchors can make both AOA and RSSI measurements for the conventional AOA/RSSI positioning. To evaluate the accuracy of different localization methods, we use the expectation of RMSE, defined as

(22) $$RMSE=\sqrt{E\left\{\frac{1}{M}\sum_{m=1}^{M}{\left(\Delta r_{m}\right)}^{2}\right\}},$$

where E{.} stands for expectation over Monte Carlo simulations, and $\Delta r_{m}=\sqrt{{\left(x_{mx}-{\hat{x}}_{mx}\right)}^{2}+{\left(x_{my}-{\hat{x}}_{my}\right)}^{2}}$ is the distance from the estimated location to the real location of the *m*-th agent. In the following simulations, 1000 Monte Carlo runs will be performed and 2000 agents will be estimated in each run. Since anchors are assumed to be deployed by UAVs, the air-to-ground channel model in the urban area evaluated in [28] is adopted in this paper. As shown in [28], with the transmitted signal frequencies from 1 GHz to 2 GHz, the PLE range is from 3.76 to 4.77. Further, we assume that all anchors have fixed positions so that the RSSI values are obtained after averaging many measurements to reduce the errors caused by multipath and shadowing. Therefore, without loss of generality, the averaged PLE γ=4 is adopted at all anchors for simulations. The log-normal shadowing effect on RSSI at each anchor is generated independently in each iteration and dynamically for different agents. However, the log-normal shadowing variance $\sigma_{n_{i}}$ is assumed to be the same at all anchors. The angle estimation noise variances $\sigma_{m_{i}}$ are set to be the same at all anchors in case nAOA/nRSSI is used.

### 4.2. Impact of AOA and RSSI Measurement Errors

We firstly evaluate the impact of angle estimation errors on the performance of the above localization methods. In this simulation, the AOA estimation error $\sigma_{m_{i}}$ is set in the range of 0to10 degrees, while the log-normal shadowing is $\sigma_{n_{i}}=0$ dB.

**Figure 4:**
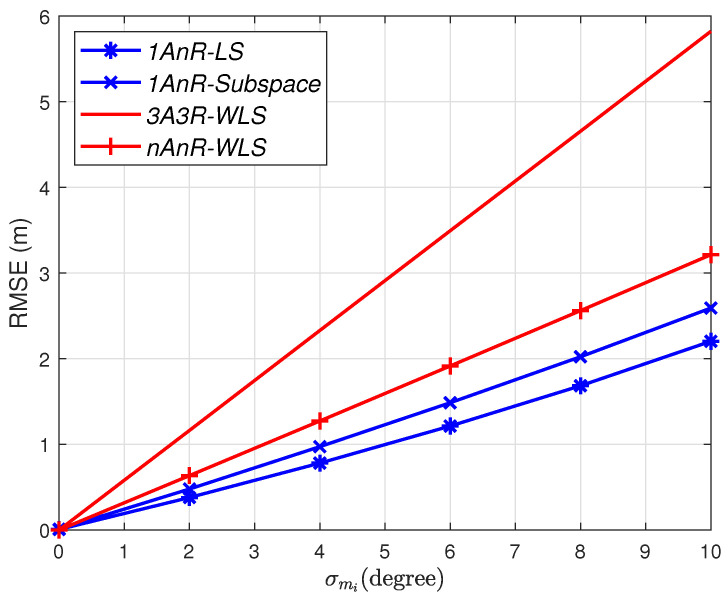
Root mean square error (RMSE) versus AOA estimation errors; $\sigma_{n_{i}}=0$ dB.

Figure 4 shows the RMSE obtained by different methods with N=10 anchors. We can see that—if perfect ranging measurement is obtained, i.e., $\sigma_{n_{i}}=0$ dB—AOA estimation errors cause *nAnR-WLS* to be worse than both *1AnR* algorithms. The reason is that when AOA estimations suffer from significant errors, such as under a non-line-of-sight (NLOS) environment, the more AOA information is involved and the worse the level of precision that can be obtained.

**Figure 5:**
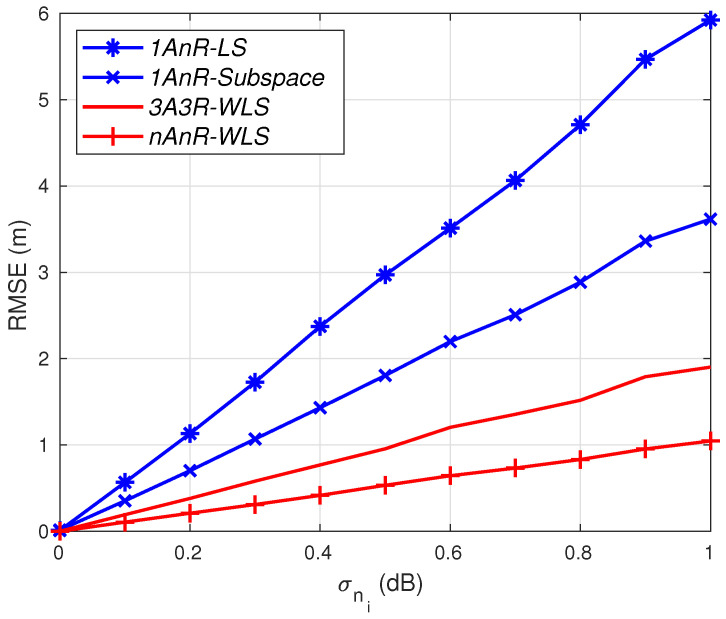
RMSE versus RSSI estimation errors; $\sigma_{m_{i}}=0$ degrees.

We then evaluate the impact of RSSI measurement errors on the performance of those methods. In the second simulation, $\sigma_{n_{i}}$ is set in the range from 0.1 to 1 dB, while $\sigma_{m_{i}}$ is 0 degrees. Over the same number of runs and agents, the RMSE is presented in Figure 5. We can see that *1AnR-LS* is much more sensitive to the range estimation error than the subspace one.

In the last two simulations, the RMSE is calculated under both AOA and RSSI measurement errors. The RMSEs are also compared to the Cramer–Rao lower bounds (CRLB) for both conventional nAOA/nRSSI and proposed 1AOA/nRSSI estimators, denoted by *CRLB-nAnR* and *CRLB-1AnR*, respectively. Detailed derivations of these bounds are presented in Appendix A.

**Figure 6:**
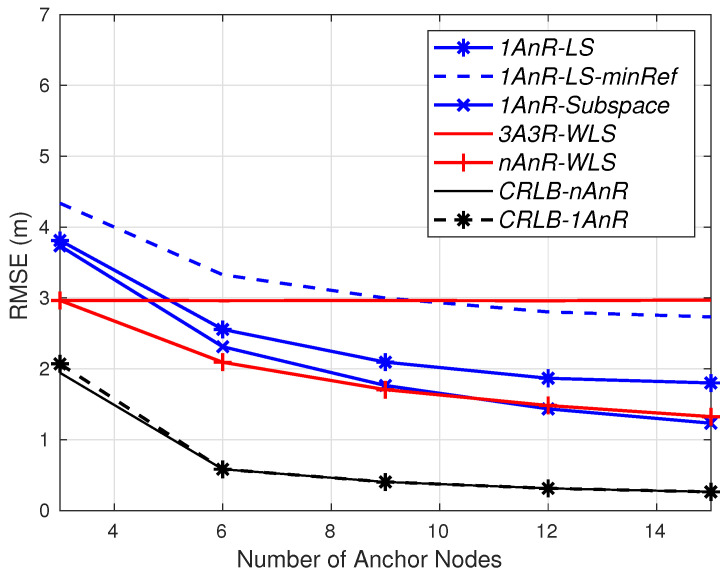
RMSE for $\sigma_{n_{i}}=0.3$ dB, $\sigma_{m_{i}}=5$ degrees.

**Figure 7:**
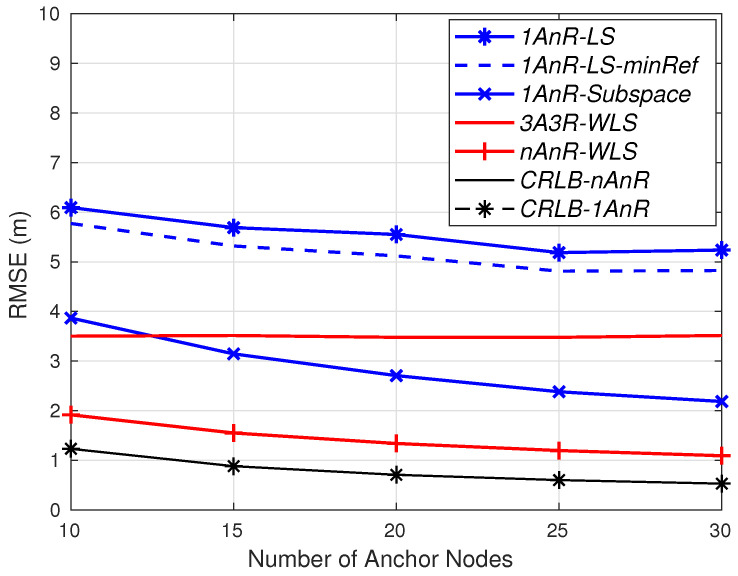
RMSE for $\sigma_{n_{i}}=1$ dB, $\sigma_{m_{i}}=5$ degrees.

Considering the AOA error $\sigma_{m_{i}}=5$ degrees and the number of available anchors from 3 onward, the RMSEs for $\sigma_{n_{i}}=0.3$ dB and $\sigma_{n_{i}}=1$ dB are depicted in Figure 6 and Figure 7, respectively. Under a small shadowing effect ($\sigma_{n_{i}}=0.3$ dB), the two 1AOA/nRSSI methods can achieve the same accuracy as the 3AOA/3RSSI approach with a slightly higher number of simple anchors. In particular, both *1AnR* methods surpass the accuracy of *3A3R-WLS* with 5 anchors. When more than 10 simple anchors are employed, the subspace method outperforms the fully *nAnR-WLS*. It is worth noting that the fully *3A3R-WLS* and *nAnR-WLS* always require an antenna array and a more complex signal processing capability (thus power supply) at each anchor, while the *1AnR* methods only require them at the master anchor.

The dashed blue line is *1AnR-LS-minRef*, in which the reference anchor is chosen to have the shortest estimated range to the agent. In Figure 6, *1AnR-LS* with the master anchor (at the origin) as the reference is superior to *1AnR-LS-minRef*. However, in case of a more severe shadowing environment in Figure 7, *1AnR-LS-minRef* outperforms *1AnR-LS*. The reason is that, in the former case, the master anchor has a significant impact on the performance of the LS method as it is used three times compared to a small total number of anchors, as explained in Section 3. Meanwhile, in the latter case with more severe distance measurement errors, the reference anchor found from a higher number of simple available anchors is more reliable than the master one.

Under the more severe shadowing effect ($\sigma_{n_{i}}=1$ dB) as shown in Figure 7, the performance of the 1AOA/nRSSI LS estimator is far worse than the subspace counterpart. *1AnR-Subspace* needs up to 12 simple anchors to achieve the same accuracy as *3A3R-WLS*; while in case of *1AnR-LS*, the required number of simple anchors will be much higher (N>30) to reach the accuracy of *3A3R-WLS*.

These simulation results confirm that the proposed *1AnR-Subspace* and *1AnR-LS* perform well in low-shadowing-effect environments with a slightly higher number of simple anchors (N=5) to estimate the agents’ location. In practice, one quick deployment method for these simple anchors is to fly some UAVs in certain trajectories through the expected anchor positions. The deployment of UAVs also allows the links from the anchors to the ground agents to have low shadowing. Meanwhile, in more severe shadowing environments, *1AnR-Subspace* is more efficient as it requires a significantly lower number of anchors compared to *1AnR-LS*, while its computational complexity is slightly higher than the LS counterpart.

It is worth noting that in both cases of the shadowing effects, *CRLB-nAnR* and *CRLB-1AnR* are almost the same. This means that the 1AOA/nRSSI estimator can ultimately achieve the same precision as the conventional nAOA/nRSSI—for example, when the number of anchors in both methods is very large—while the proposed method significantly reduces both hardware and computational complexities.

**Figure 8:**
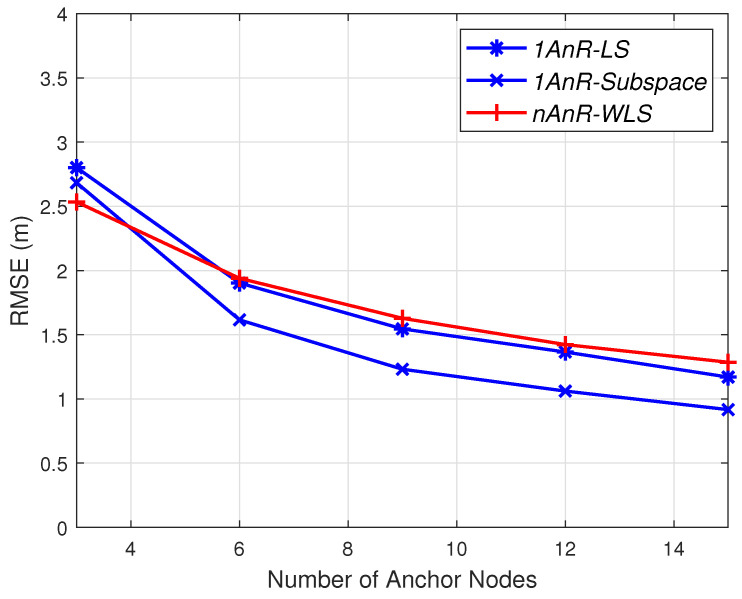
RMSE for $\sigma_{n_{i}}=0.3$ dB, $\sigma_{m_{i}}=5$ degrees, with the master anchor at the centre of the area.

These simulation results imply that there is still room to improve the performance of the 1AOA/nRSSI. For example, more complicated algorithms may be used to achieve the same accuracy as the nAOA/nRSSI method, while the additional number of anchors remains small. In addition, as observed from Figure 6 and Figure 7, the accuracy can be improved by selecting an optimal position of the master anchor. As discussed in Section 2.2, the anchor that has the closest distance to the agent should be emphasized because it has smaller estimation errors. However, since all anchors are deployed at their fixed positions, while targets can appear anywhere in the area, it is impossible to select the master anchor with the shortest range to every target. To demonstrate the advantage of the optimal master anchor placement, we simulate a simple case of the master anchor placed at the center of the area. Since targets are uniformly distributed in the area, the average distance from the targets in the area to the master anchor is the shortest. Figure 8 depicts the RMSE for the same setup in Figure 6 but the master anchor is located at the center of the area, rather than at the origin. Clearly, under the same level of measurement errors, both 1AnR methods outperform the *nAnR-WLS* one when N≥6 compared to N≥10, as shown in Figure 6.

Furthermore, the accuracy of the 1AOA/nRSSI estimator can be improved if the master anchor provides a more accurate AOA estimation. This means that instead of equipping all anchors with AOA measurements as in the conventional nAOA/nRSSI method, we can invest in the master anchor with a significantly large antenna array and sufficient signal processing capability, so that the 1AOA/nRSSI estimator has superior angle information than that in the nAOA/nRSSI. Hence, unbalanced hybrid AOA/RSSI localization allows a more flexible deployment of WSN.

## 5. Conclusions

This paper proposes an unbalanced hybrid AOA/RSSI localization method to simplify the WSN. By requiring only one AOA information to combine with RSSI measurements, the large antenna array and complicated signal processing required at all anchors nodes are not necessary except for one master anchor. Two different 1AOA/nRSSI localization methods are presented and evaluated under different scenarios. The simulation results show that the 1AOA/nRSSI LS estimator is suitable when more accurate RSSI measurements are available, while the subspace method is more suitable in a shadowing environment with an acceptable increase in the number of anchors and computational complexity. Possible future directions include improving the precision of 1AOA/nRSSI localization algorithms using different approaches listed in Section 4 and adopting an analog least mean square loop presented in [29,30,31,32] for a more accurate ranging estimation. Additionally, we might consider orthogonal frequency division multiplexing (OFDM) for correlated multipath fading channels [33] in the localization and positioning contexts.

## Abbreviations

The following abbreviations are used in this manuscript:

AOA	Angle-of-Arrival
CRLB	Cramer-Rao Lower Bound
FIM	Fisher Information Matrix
GPS	Global Positioning System
LS	Least Square
NLOS	Non-Line-of-sight
ML	Maximum Likelihood
OFDM	Orthogonal-Frequency Division Multiplexing
PDF	Probability Density Function
PLE	Path-loss Exponent
RMSE	Root Mean Square Error
RSSI	Received Signal Strength Indicator
TOA	Time-of-Arrival
TDOA	Time-Different-of-Arrival
TOF	Time-of-Flight
UAV	Unmanned Aerial Vehicle
WLS	Weighted Least Square
WSN	Wireless Sensor Network

## Appendix A. CRLB Derivation

Cramer-Rao Lower Bound (CRLB) is an important benchmark to compare different unbiased estimators. CRLB is calculated from a Fisher information matrix (FIM) which contains the expected value of the observed ranging information. Define a generalized measurement vector θ as

(A1) θ=f(x)+n

where f(.) is a nonlinear function of the agents’ positions x and n is an additive zero-mean noise vector. Since hybrid AOA/RSSI information is in use, θ includes both RSSI and AOA measurements, i.e., $\theta ={\left[\theta_{rss,1},\ldots ,\theta_{rss,N},\alpha_{1},\ldots ,\alpha_{L}\right]}^{T}$ where L=N for the case of the conventional nAOA/nRSSI and L=1 for the case of 1AOA/nRSSI. $\theta_{rss,i},i=1,\ldots ,N$, is the RSSI represented in the Equation (A1) form, i.e.,

(A2) $$\theta_{rss,i}=P_{i}-P_{0}=-10\gamma \log_{10}\left(d_{i}\right)+n_{i}.$$

Substituting $d_{i}=\sqrt{{\left(x_{x}-a_{ix}\right)}^{2}+{\left(x_{y}-a_{iy}\right)}^{2}}$ into Equation (A2) and utilizing $\alpha_{i}$ calculated from the second equation in Equation (1), f(x) is written as

(A3) $$f\left(x\right)=\left[\begin{array}{c} -10\gamma \log_{10}\left(\sqrt{{\left(x_{x}-a_{1x}\right)}^{2}+{\left(x_{y}-a_{1y}\right)}^{2}}\right)\\ \vdots \\ -10\gamma \log_{10}\left(\sqrt{{\left(x_{x}-a_{Nx}\right)}^{2}+{\left(x_{y}-a_{Ny}\right)}^{2}}\right)\\ \tan^{-1}\left(\frac{x_{y}-a_{1y}}{x_{x}-a_{1x}}\right)\\ \vdots \\ \tan^{-1}\left(\frac{x_{y}-a_{Ly}}{x_{x}-a_{Lx}}\right) \end{array}\right].$$

The FIM, denoted by I(x), is the expected values of the second-order derivatives of the logarithm of the measurement probability density function (PDF) with respect to x, i.e.,

(A4) $$I\left(x\right)=E\left\{\frac{\partial^{2}\ln p\left(\theta \right)}{\partial x\partial x^{T}}\right\}$$

where p(θ) is the PDF of the measurements θ. As proved in [34], when n is zero-mean Gaussian distributed, I(x) can be also calculated by

(A5) $$I\left(x\right)={\left[\frac{\partial f(x)}{\partial x}\right]}^{T}C^{-1}\left[\frac{\partial f(x)}{\partial x}\right]$$

where $C=\mathrm{diag}\{\sigma_{n_{1}}^{2},\ldots ,\sigma_{n_{N}}^{2},\sigma_{m_{1}}^{2},\ldots ,\sigma_{m_{L}}^{2}\}$ is the covariance matrix of the noise vector n.

Taking partial derivations of f(x) over the two dimensions *x* and *y*, denoted as $\frac{\partial f(x)}{\partial x_{x}}$ and $\frac{\partial f(x)}{\partial x_{y}}$ respectively, we have

(A6) $$\begin{array}{cc} \frac{\partial f(x)}{\partial x_{x}} & ={\left[-\eta \frac{x_{x}-a_{1x}}{d_{1}},\ldots ,-\eta \frac{x_{x}-a_{Nx}}{d_{N}},-\frac{x_{y}-a_{1y}}{d_{1}^{2}},\ldots ,-\frac{x_{y}-a_{Ny}}{d_{N}^{2}}\right]}^{T}\\ \frac{\partial f(x)}{\partial x_{y}} & ={\left[-\eta \frac{x_{y}-a_{1y}}{d_{1}},\ldots ,-\eta \frac{x_{y}-a_{Ny}}{d_{N}},\frac{x_{x}-a_{1x}}{d_{1}^{2}},\ldots ,\frac{x_{x}-a_{Nx}}{d_{N}^{2}}\right]}^{T} \end{array}$$

where $\eta =\frac{10\gamma }{\ln10}$. Hence, I(x) is found:

(A7) $$I\left(x\right)=\left[\begin{array}{cc} \sum_{i=1}^{N}\frac{\eta^{2}{\left(x_{x}-a_{ix}\right)}^{2}}{\sigma_{n_{i}}^{2}d_{i}^{2}}+\sum_{j=1}^{L}\frac{{\left(x_{y}-a_{jy}\right)}^{2}}{\sigma_{m_{j}}^{2}d_{j}^{4}} & \sum_{i=1}^{N}\frac{\eta^{2}\left(x_{x}-a_{ix}\right)\left(x_{y}-a_{iy}\right)}{\sigma_{n_{i}}^{2}d_{i}^{2}}-\sum_{j=1}^{L}\frac{\left(x_{x}-a_{jx}\right)\left(x_{y}-a_{jy}\right)}{\sigma_{m_{j}}^{2}d_{j}^{4}}\\ \sum_{i=1}^{N}\frac{\eta^{2}\left(x_{x}-a_{ix}\right)\left(x_{y}-a_{iy}\right)}{\sigma_{n_{i}}^{2}d_{i}^{2}}-\sum_{j=1}^{L}\frac{\left(x_{x}-a_{jx}\right)\left(x_{y}-a_{jy}\right)}{\sigma_{m_{j}}^{2}d_{j}^{4}} & \sum_{i=1}^{N}\frac{\eta^{2}{\left(x_{y}-a_{iy}\right)}^{2}}{\sigma_{n_{i}}^{2}d_{i}^{2}}+\sum_{j=1}^{L}\frac{{\left(x_{x}-a_{jx}\right)}^{2}}{\sigma_{m_{j}}^{2}d_{j}^{4}} \end{array}\right].$$

Finally, the CRLB is obtained by

(A8) $$CRLB=\sqrt{E\left\{\frac{1}{M}\sum_{m=1}^{M}\frac{{\left[I^{-1}\left(x\right)\right]}_{1,1}^{\left(m\right)}+{\left[I^{-1}\left(x\right)\right]}_{2,2}^{\left(m\right)}}{2}\right\}}$$

where E{.} denotes expectation over all Monte Carlo iterations.
